# The bumpy road to recovery: older adults’ experiences during the first year after hip replacement surgery - a longitudinal qualitative study

**DOI:** 10.1186/s12877-025-06155-6

**Published:** 2025-07-02

**Authors:** Lina Bergman, Amina Guenna Holmgren, Ulrica Nilsson, Anahita Amirpour, Helen Conte, Jeanette Eckerblad

**Affiliations:** Department of Neurobiology, Care Sciences and Society, Karolinska Institutet, Alfred Nobels Allé 23, C3| 141 83 Hudding, Huddinge, Sweden

**Keywords:** Longitudinal qualitative study, Older adults, Postoperative recovery, Postoperative nursing

## Abstract

**Background:**

Half of the older adult population worldwide is expected to undergo surgery at least once in their lifetime, with hip and knee replacements being among the most common procedures. Recovery after surgery is particularly difficult for older adults because of age-related physiological changes, an increased risk of complications, and the need for self-management at home following brief hospital stays. Emotional challenges such as fear, anxiety, and loss of independence further impact recovery, yet these aspects are often overlooked in clinical care. Currently, there is a lack of understanding of how older adults experience postoperative recovery after hip replacement surgery over time.

**Objective:**

To explore the experiences of recovery over time among older adults during the first year after hip replacement surgery.

**Methods:**

This descriptive longitudinal qualitative study included 30 older adults scheduled for hip replacement surgery at a university hospital in Sweden. Semistructured interviews were conducted twice, at fourteen days and one year after surgery. The data were analyzed using qualitative content analysis, following a Pattern-Oriented Longitudinal approach to inductively describe experiences over time.

**Results:**

The analysis resulted in one overarching theme: “the bumpy road to recovery” and three categories: physical challenges post-surgery, cognitive and emotional changes after surgery, and social and daily life adjustments. Recovery involves a balance between resting, managing symptoms, and regaining strength. For many, recovery involves physical, emotional, and cognitive challenges that feel discouraged.

**Conclusion:**

This study highlights the complex and individualized recovery process after hip replacement surgery. The participants navigated physical, cognitive, emotional, and social challenges and reached a new stable state after surgery. Balancing rest and activity, managing symptoms, and maintaining social connections are key factors for successful recovery. Preoperative education and support from healthcare providers and family members are vital in improving the recovery process for older adults.

**Supplementary Information:**

The online version contains supplementary material available at 10.1186/s12877-025-06155-6.

## Background

Globally, it is estimated that half of the older adult population will undergo at least one surgical procedure in their lifetime [1]. The number of older adults undergoing surgery is increasing more rapidly than that of the general population [2]. For older adults, surgery is a significant event since they are more prone to postoperative complications [3]. Hospital stays following surgery are typically short, and efforts to reduce these stays are not always beneficial for older adults [4]. Consequently, most of the recovery process occurs at home, requiring older adults to manage their symptoms independently[5]. The postoperative recovery process can be particularly challenging [6] for older adults because of the effects of aging on the organ system [7, 8]. Therefore, it is crucial to explore how postoperative recovery is experienced and how older adults cope with these changes over time.

Postoperative recovery is a challenging period of transition, involving both an individual process and a transformative journey [9, 10]. This journey involves regaining normal functions and achieving optimal well-being [5, 6], reaching a new stable state that may be the same, better or worse than preoperatively [10]. It has also been defined as ‘getting back to normal‘[11] or “to be as before” [10]. The postoperative recovery journey includes physical, emotional, social, and habitual aspects [6, 10, 12] and can be divided into different phases: early, intermediate, late, and long-term recovery [5, 13] as well as a pre-recovery phase [5, 14, 15]. Early and intermediate recovery occurs before discharge from the hospital and includes normalizing coordination and physiological function to achieve “home readiness” [5]. Late recovery begins upon discharge and continues until the patient regains usual function and activity, which can take hours to days [13]. Long-term recovery often takes three to six months to restore functional and cognitive abilities [5]. Recovery time varies from days to months, with older individuals generally experiencing slower recovery than younger people do [3, 11]. This is particularly relevant for older individuals with physical limitations [11], as the likelihood of postoperative complications is greater for medical reasons than for surgical reasons [8, 16]. Some may never fully recover to their previous level of functioning [11]. Apart from postoperative pain, a significant aspect of the postoperative recovery period is the temporary loss of independence [11, 12, 14]. Additionally, emotions such as fear, anxiety, and powerlessness [11, 12, 17], as well as fatigue [9, 12, 18, 19], are commonly reported. These aspects of postoperative recovery are rarely discussed among healthcare professionals and are communicated to patients or their relatives. Patients who experience postoperative symptoms and complications may be taken aback by their impact on recovery[15, 19]. These complications can affect psychosocial functioning and the ability to participate in daily activities [19], leading to feelings of helplessness and uncertainty [15]. Therefore, informing patients about common complications and symptoms and providing and explaining their potential impact on recovery are crucial [15].

Hip and knee replacement surgeries are among the most common procedures, accounting for 18.8% of all surgeries among older adults [20]. The number of individuals undergoing hip replacement surgery has significantly increased over the past decade, and this trend is expected to continue as the population ages [21]. Patients’ experiences after hip replacement surgery have previously been described [4, 22, 23], with a focus mostly on the early and intermediate phases of recovery. During these initial phases of recovery, the commonly reported symptoms are pain and fatigue [4, 22]. Patients also describe improving and regaining physical function as important issues [4]. However, few studies have investigated late- and long-term recovery among older adults. One study described experiences of pain and rehabilitation three months after surgery, focusing primarily on the physical dimension of recovery [23], but studies investigating emotional, social, and habitual dimensions of recovery and the recovery trajectory over time are lacking. Currently, there is a limited understanding of how older adults experience postoperative recovery after hip replacement surgery over time. Addressing this knowledge gap is essential to effectively support this population during their late and long-term postoperative recovery.

## Methods

### Aim

To explore the experiences of recovery over time among older adults during the first year after hip replacement surgery.

### Design

This study used a descriptive longitudinal qualitative design [24]. The data collected were part of a larger mixed-methods study [25]. The reporting of findings followed the Consolidated Criteria for Reporting Qualitative Research (COREQ) guidelines (Supplementary file 1) [26].

### Setting and sample

The study was conducted at a university hospital in Stockholm, Sweden. Patients aged 60 years or older who were scheduled for hip replacement surgery were eligible for inclusion. The exclusion criteria, aligned with those of the larger mixed-methods study, included significant hearing or visual impairment, a Mini-Mental State Examination (MMSE) score below 23, [27] neurological disease, dependence on antidepressants or tranquilizers, substance misuse, recent surgery (within the previous six months), and insufficient fluency in Swedish.

An unauthorized version of the Swedish MMSE was used by the study team without permission; however, this has now been rectified with Psychological Assessment Resources (PAR). The MMSE is a copyrighted instrument and may not be used or reproduced in whole or in part, in any form or language, or by any means without written permission of PAR.

A total of forty individuals were consecutively enrolled in the study. Of these, ten declined to participate in a second interview conducted at 12 months. While participants were not required to provide a reason for withdrawal, some mentioned poor health or lack of time. However, the number of respondents and specific reasons were not systematically documented. The final dataset includes 60 interviews from the 30 participants who completed both interviews, 14 days and 1-year postoperatively.

### Data collection

Data were collected between October 2019 and November 2021 with an extension due to the COVID-19 outbreak. Potential participants were recruited during their preoperative visit, informed about the study, and invited to participate. Qualitative data were collected at two time points: 14–16 days after surgery (late recovery phase) and one year after surgery (long-term recovery phase).

Using a semistructured interview guide (Supplementary File 2), we conducted individual interviews to explore recovery, cognitive function, and overall well-being. The interview guide was developed by two authors (JE and UN), both with extensive experience in postoperative research and clinical work. Findings related to postoperative neurocognitive recovery at 14 days have been published previously [25]; however, data related to the broader aspects of recovery and experiences one year after surgery have not been reported.

The first round of interviews (median duration: 21 min, range: 10–33 min) was conducted face-to-face at the hospital. The second round of interviews, conducted by telephone with the same participants, had a median duration of 13 min (range: 8–29 min). All the interviews were carried out by the same research nurse, who had no prior relationship with the participants. The interviews were audio-recorded and transcribed verbatim. Demographic and perioperative data were extracted from the participants’ medical records.

### Data analysis

The phenomenon of interest was the participant’s recovery over time. A pattern-oriented longitudinal analysis approach (POLA) was used to inductively describe and understand changes and variations in experiences [24]. The interview data were analyzed using qualitative content analysis as outlined by Elo and Kyngäs [28].

The analysis process consisted of two stages. In the first stage, the data were structured and interpreted by four authors (AGH, LB, HC, JE), who read all 60 interviews and engaged in discussions about content, quality, and analytical methods. The dataset was divided into four parts, with each author analyzing the data of 7–8 participants (14–16 interviews). The same four authors participated in the initial coding process, starting with the first interview, identifying meaning units, organizing them into content areas, and applying the same process to the subsequent interviews. Individual experiences and changes over time were summarized for each participant. To ensure consistency, two authors (AGH and JE) reviewed and coded all 60 interviews. A follow-up meeting was held to discuss commonalities, variations and to plan the second stage of analysis.

In the second stage, two authors (AGH and JE) focused on identifying and summarizing similarities and variations in experiences across the entire dataset. Preliminary categories were created for each dataset separately. The categories from the first interview set (14 days postoperatively) were then compared with those from the second set (1-year postoperatively). When differences were identified, the coding structure was modified: categories and subcategories were added, removed, adjusted, or merged as needed to accurately reflect continuity and change over time. This iterative process ensured that the final categorization captured the range of experiences across both time points. Table 1 illustrates an example of the analysis process; however, not all subcategories identified during the analysis are represented in the table. Throughout the analysis, all the authors were engaged in ongoing discussions to refine and validate the analysis process.

**Table 1:** Example of the analysis process

Preliminary categories at 14–16 days after surgery	Preliminary categories one year after surgery	Subcategories	Categories	Theme
Affected by pain	Decreased perception of pain	Coping with pain and discomfort	Physical challenges post-surgery	The bumpy road to recovery
Memory impairment and cognitive deficits	Alleviation of cognitive and memory difficulties	Struggling with cognitive activities	Cognitive and emotional changes after surgery	
Reduced ability and willingness to engage in social activities	Resumption of social engagement	Regaining engagement in social activities	Social and daily life adjustments	

### Rigour and reflexivity

To ensure trustworthiness, multiple techniques were employed throughout the study. Investigator triangulation was used to enhance credibility and confirmability [29]: at least two authors independently analyzed each transcript and compared interpretations for similarities and differences. Any discrepancies were discussed until consensus was reached, thereby minimizing individual researcher bias and ensuring that categories were internally homogeneous and externally heterogeneous [30].

Dependability was supported by consistent data collection procedures: all interviews were conducted by the same research nurse using a semi-structured interview guide and within a defined timeframe. The analytical process closely followed the principles of the selected design and analytic approach [29].

Transferability was addressed by providing rich descriptions of the sampling strategy, eligibility criteria, data collection procedures, and analytical methods.

A reflexive approach was maintained throughout the research process. The involved researchers continuously discussed and challenged their preunderstandings and assumptions, aiming to identify and minimize potential biases. Coding and interpretation were reviewed in collaborative meetings to enhance interpretative rigor. The research team brought substantial domain expertise to the project: all six authors (LB, AGH, UN, AA, HC, JE) are registered nurses with extensive experience in postoperative care and qualitative research, and five hold a PhD.

### Ethical considerations

We followed the ethical principles of the Declaration of Helsinki and obtained ethical permit (2019–02968) from the Swedish Ethical Review Authority. All participants received written and verbal information about the study, and we obtained written informed consent prior to enrollment.

## Results

**Fig. 1:**
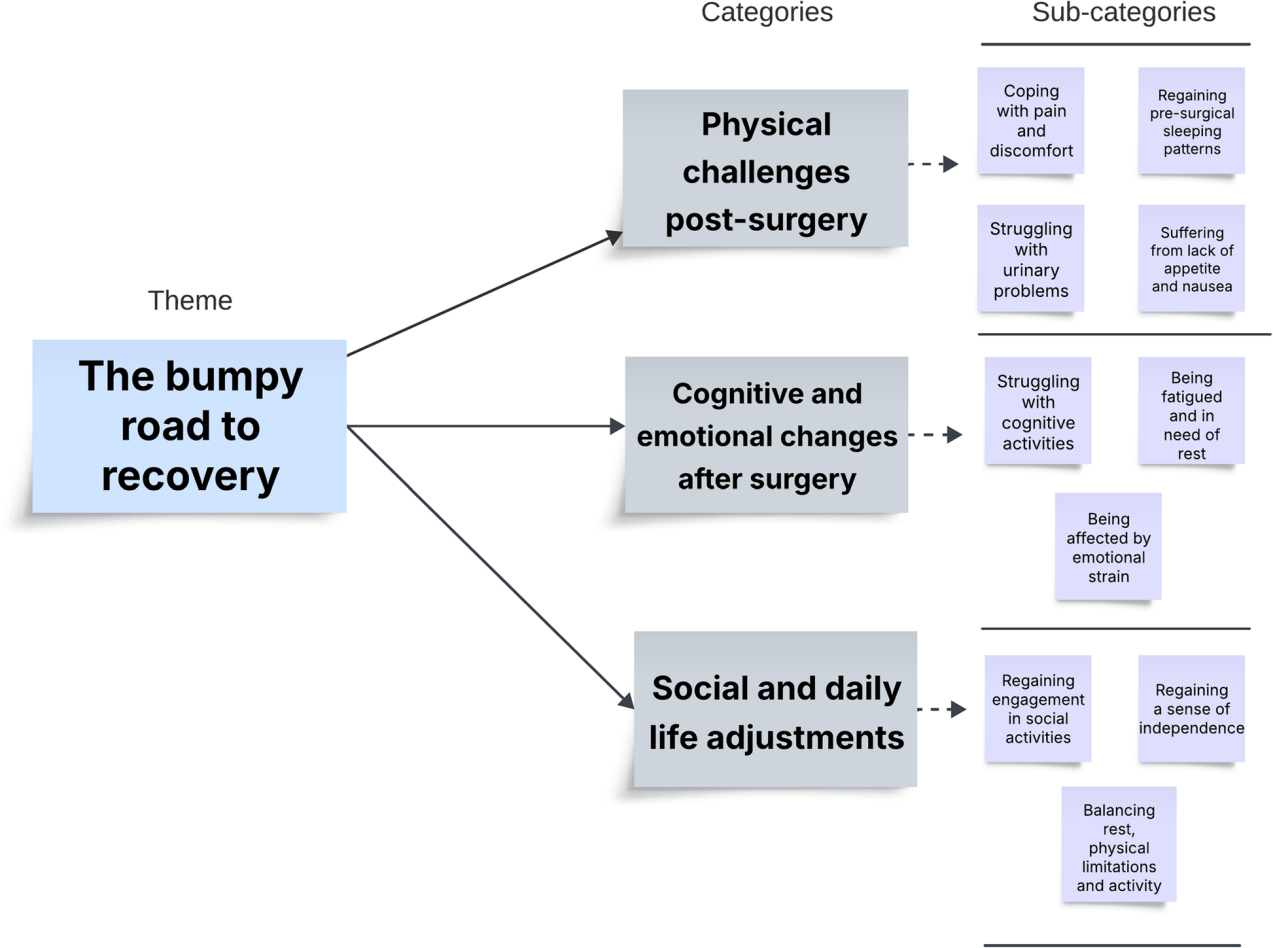
Main theme and categories from the analysis

All participants in this study were community-dwelling older adults, with a mean age of 73 years; 67% were women. For additional participant characteristics, see Table 2. The analysis revealed one overarching theme, three categories, and ten subcategories, as illustrated in Fig. 1.

**Table 2:** Participant characteristics

Characteristics	Total (*n* = 30)
Age, years	
Mean (minmax)	73.5 (60–87)
Gender, n (%)	
Male	13 (43)
Female	17 (57)
Education, n (%)	
Elementary school	9 (30)
Upper secondary school	10 (33)
Tertiary education	11 (37)
Housing, n (%)	
Apartment with elevator	12 (40)
Apartment no elevator	7 (23)
House with stairs	9 (30)
House no stairs	2 (7)
Relative, n (%)	
Spouse or partner	18 (60)
Living alone	8 (27)
Living with adult children,	2 (7)
Living alone with home care	1 (3)
Spouse and homecare	1 (3)
MMSE, preoperative	
Mean (SD)	28.6 (1.4)
Duration of anesthesia, minutes	
Mean (SD)	190.1 (38.0)
Hospital length of stay, days	
Mean (SD)	1.5 (0.6)

### The bumpy road to recovery

The participants’ experiences with the recovery process suggest that it could be characterized as a journey. Recovery involves a balance between resting, managing symptoms, and regaining strength. Some described the road as a journey toward full recovery, which was often not straightforward, frequently bumpy, and influenced by factors such as the normal trajectory of aging, previous experiences, and other life events. For most participants, recovery was not just about healing from surgery; it also meant coping with the effects of aging. A combination of physical, emotional, and cognitive problems creates a challenging recovery process for many, leaving them to feel stuck and discouraged. These conditions often had a greater impact on their daily lives than did the surgery itself, making many patients feel that their recovery was incomplete. However, several factors facilitate successful recovery, such as physical activity, maintaining an active lifestyle, and engaging in cognitive and social activities.

### Physical challenges post-surgery

The participants described a variety of physical symptoms that occurred during their recovery period, predominantly during their late recovery phase. Physical limitations, whether caused by the surgery itself or by postoperative symptoms, impact functional ability and delay the return to daily life for many participants. The participants described the period directly following surgery as a time of trying to regain strength through rest and waiting. For the majority of patients, there was a gradual decrease in physical postoperative symptoms, and by the one-year mark, only a few still experienced physical challenges.

#### Coping with pain and discomfort

The participants reported varying experiences of postoperative pain during their late recovery phase. Some experienced less pain than expected, whereas others faced significant pain and responded poorly to analgesics. The pain limited their mobility and independence and was described as a nuisance. Although the experience of pain-related limitations decreased during the first year, it persisted to some extent for some participants. Many were pain-free after one year, but those with ongoing pain felt disappointed with their recovery. These participants had discontinued their prescribed analgesics and chose not to seek medical assistance, preferring to wait and see if the pain would resolve naturally.

The participants’ varying pain experiences influenced their use of analgesics. Those who experienced pain often took short-acting oxycodone for breakthrough pain. Participants who did not experience severe pain or were not significantly bothered by it quickly reduced their use of analgesics, particularly oxycodone, sometimes even before discharge from the hospital. However, some participants who experienced severe pain in the late recovery phase still chose to decrease the use of analgesics. Several factors, including fear of the medication, concerns about addiction, and potential side effects, influenced their decision to reduce the medication. Oxycodone was regarded as a last resort, used only for severe pain, and was often compared to alcohol due to its hangover-like effects.

” But then after the first few days, I felt ‘hungover’ from these morphine and paracetamol pills, so I stopped taking them after three days.” (Participant 44, 14 days post-surgery).

The participants attributed symptoms such as fatigue, dizziness, nausea, nightmares, and gastrointestinal issues to oxycodone side effects, often preferring to endure the pain rather than deal with these adverse effects.

” I think I should have stopped taking these medications much earlier. Then my stomach would have been fine.” (Participant 25, 14 days post-surgery).

#### Regaining pre-surgical sleeping patterns

In the late recovery phase, participants reported difficulty sleeping due to pain following surgery, movement restrictions, or general inactivity.

”That I have to lie on my back. It’s really tough. I usually like sleeping on my back and turning over. But now I can’t turn over, I’m not allowed to.” (Participant 36, 14 days post-surgery)”.

Additionally, several participants mentioned that they had difficulty sleeping before the surgery and that these problems intensified in the late recovery phase. This sleep deprivation led to frustration and anger in some participants, whereas others were more accepting of the situation.

To cope with their sleeping problems, the participants took short naps during the day and were careful not to nap too long to avoid further disrupting their nighttime sleep. Analgesics were also used to aid sleep and reduce anxiety. The impact of sleep deprivation varied among the participants; some reported that managing their emotions and daily activities was challenging due to a lack of rest, whereas others adapted more easily. Despite these challenges, one year after surgery, most participants resumed their pre-surgery sleep patterns, indicating a return to their normal routines and an improvement in their overall well-being.

“I have trouble sleeping, I take sleeping pills, but I did that before the surgery too, so there’s no difference there.” (Participant 18, 1-year post-surgery).

#### Struggling with urinary problems

The participants experienced difficulties with urination in the initial late recovery phase. Previous urinary problems among the participants worsened after surgery, with some struggling with urinary retention and nocturia. These issues were predominantly reported by the men in the study. The difficulties resulted in some patients being discharged from the hospital with a urinary catheter, which was later removed at their local healthcare clinic. Additionally, several participants experienced nocturia, a condition that worsened postoperatively, causing frequent bathroom visits that disrupted their sleep.

” Well, to urinate that is probably the most… It’s not completely under control.” (Participant 17, 14 days post-surgery).

For some, these issues subsided over time, bringing relief and a return to normalcy. However, others continued to experience similar problems even one year after surgery, indicating a prolonged impact on their quality of life.

#### Suffering from lack of appetite and nausea

Most participants experienced a reduced appetite in the initial days following surgery, and many were troubled by persistent nausea. This combination of nausea and lack of appetite made it particularly difficult for them to prepare meals. Those who relied on home care services highlighted this issue even more, noting that the food provided was often unappetizing and not served at times when they were hungry. This mismatch in meal timing and quality further contributed to their reduced appetite.

The participants expressed frustration and discomfort, as the lack of appealing food options and the inconvenience of mealtimes added to their overall stress. Despite these challenges, most participants noted that their appetite generally returned within a week or two postoperatively.

” But I didn’t have much of an appetite during the first three or four days.” (Participant 17, 14 days post-surgery).

### Cognitive and emotional changes after surgery

In addition to physical challenges, participants experienced cognitive and emotional changes after surgery that affected their health and overall well-being. These symptoms impact their life in various ways. Some described an increased dependency on others when their cognitive abilities decreased, whereas others noted how fatigue hindered their ability to initiate activities. Most participants regained their cognitive function and the ability to take initiative within their long-term recovery phase, leading to a marked improvement in their overall well-being.

#### Struggling with cognitive activities

Some participants described concentration problems postoperatively. They reported reading or solving puzzles like crosswords and sudoku challenging, which they easily managed before surgery. During the long-term recovery phase, most of the participants experienced improvement, but some still reported that cognitive activities were challenging.

” And doing crosswords after the surgery, I tried. I always do them during breakfast. But I didn’t understand anything.” (Participant 19, 14 days post-surgery).

Several participants experienced memory loss during their long-term recovery phase. They described a noticeable decline in working memory, which made it challenging to remember names, places, or even if they had taken their medication. Participants often recognized memory loss as a result of aging or the analgesics prescribed after surgery. Many believe that memory issues, including difficulty with both short- and long-term memory, could be related to the surgery itself. These memory issues were frustrating and made them feel helpless during their recovery.

” Then that it slowly goes downhill, that it gets worse and worse, you are aware of that. Because you’re not 20 years old anymore.” (Participant 42, 1-year post-surgery).

Moreover, some participants described experiencing initial postoperative apathy and depression. They felt unable or unwilling to initiate activities or even get out of bed. These feelings gradually improved over time, with significant progress noticeable after the late recovery phase concluded.

#### Being fatigued and in need of rest

In the first few weeks after surgery, the participants described feeling fatigued and in need of rest. Daytime fatigue was particularly troublesome, especially during the late recovery phase. Many participants only got out of bed to use the bathroom or occasionally to get something to eat. They spent a considerable amount of time watching TV and attempting to read books. Some participants found it difficult to read and had trouble making sense of the text, preferring to watch TV because it required less effort. After the late recovery phase, fatigue decreased for some participants, and by the one-year follow-up, most had returned to their preoperative state.

” Yes, I have recovered, but I think it took a long time to recover from that. (…) It was only about six months ago that I started to feel like I was getting my strength back. So, I’ve felt a bit off (…) Yes, but I felt weak, had shortness of breath, and lacked energy, you could say. And then the pain from my hip also increased a bit, and I also got pain in my back. So, a lot was happening with my body.” (Participant 7, 1-year post-surgery).

Those who continued to experience fatigue in their long-term recovery also suffered from comorbidities or other issues that affected their energy levels.

#### Being affected by emotional strain

In addition to experiencing fatigue, the participants also experienced mood swings. Their mood could change quickly, with anger and frustration directed at their next of kin, whom they felt did not understand their situation. Anger could also be directed at strangers, such as a taxi driver they perceived to be driving too fast.

” At another time I might have kept quiet, but I was so angry, and he was driving so fast, so it was uncomfortable to ride with him.” (Participant 13, 14 days post-surgery).

The participants did not recognize themselves during these states, which caused them anxiety afterward, especially if they had been irritable, unkind, and demanding to people close to them. Some were still bothered by guilt one year after the surgery.

### Social and daily life adjustments

Many participants adjusted their social lives during their recovery period. Some anticipated temporary physical dependence, whereas others were surprised by the need to reduce their engagement in social activities. Participants with active social and physical lifestyles were more determined to regain their pre-surgical state, resulting in a quicker recovery process. For others, the combination of surgery and preexisting conditions makes recovery a slow and challenging process.

#### Regaining engagement in social activities

In the initial late recovery phase, participants experienced a significant drop in energy levels, severely impacting their ability to engage in social activities. Many canceled visits or postponed meetings with family and friends, frequently citing fatigue and exhaustion as the primary reasons.

”Friends called, and I told them I didn’t have the energy to have themover.” (Participant 13, 14 days post-surgery).

During their late recovery, most social interactions were limited tophone calls and emails, as in-person gatherings felt too physicallydemanding. Despite these challenges, some participants with priorcommitments to hobbies or volunteer work demonstrated a strongdetermination to resume their activities shortly after surgery, indicatinga desire to return to normal routines. Participants with active social livesor work obligations often reintegrated into these roles more quickly, andsome even returned to work within a week after their surgery.

”I still work. Not the first week, but the second week I made somevisits to some construction sites.” (Participant 44, 14 days post-surgery).

For most participants, late and long-term recovery took place during the COVID-19 pandemic, a period marked by strict social distancing guidelines in Sweden, particularly for individuals over 65 years of age. As a result, participants followed these COVID-19-related restrictions, drastically reducing in-person interactions. This period of enforced isolation deepened feelings of social disconnection, as many described a profound longing for face‒to‒face interactions that remained unmet due to safety precautions. Even as physical recovery progressed, the combination of limited social engagement and pandemic restrictions contributed to an enduring sense of isolation.

#### Regaining a sense of independence

The surgery also resulted in a temporary period of dependence on others, significantly disrupting participants’ daily routines. During the late recovery phase, many required assistance with essential tasks such as shopping, cooking, and cleaning. This support primarily came from partners or children, who took on caregiving roles to ease the burden during this time.

” I have a husband who supported and helped me, and I also had a lot of assistive devices that helped me, like crutches and a toilet chair and such. It works well.” (Participant 13, 14 days post-surgery).

For some participants, the need for assistance was more pronounced. In cases where family support was unavailable or insufficient, home care services became a vital resource. As participants regained mobility over time, their reliance on others decreased, and their ability to manage daily activities independently improved. In their long-term recovery, most participants regained a sense of independence and no longer required home care services.

” And I don´t have home care services. It stopped last fall when I was able to walk on my own.” (Participant 43, 1-year post-surgery).

However, a few still relied on relatives for occasional support with tasks like shopping or cleaning.

#### Balancing rest, physical limitations and activity

In the initial late recovery phase, participants faced significant challenges managing daily tasks due to physical limitations. These restrictions, such as reduced movement and weight-bearing on the operated leg, were particularly frustrating for those who had been active before surgery.

” I took four or five steps and then I was completely exhausted. Yes, and then I had to sit down and rest, and then it was four more steps to get to the kitchen. (Participant 10, 1-year post-surgery)

The participants adapted differently on the basis of their energy levels and pain. Some focused on rest, whereas others prioritized exercise despite their limitations. Physiotherapists provided guidance on exercise, but those who had been active prior to surgery often found these programs too basic and desired more advanced routines.

” But I feel that what was written on paper (training instructions) was too easy. So, I did more than what was written on paper.” (Participant 37, 1-year post-surgery).

For these individuals, physical activity helped reduce stiffness, manage pain, and foster a sense of progress. On the other hand, participants who had been less active before surgery found the exercises difficult to follow. Fatigue, joint pain, and mobility issues often prevented them from performing rehabilitation exercises or performing daily tasks. The participants also reported new physical symptoms or worsening of existing conditions, which made their recovery more difficult. Health issues that existed before surgery also complicated the recovery process. For example, worsening arthritis limited their ability to stay active and added to their fatigue. These conditions often had a bigger impact on their daily lives than the hip surgery itself.

Recovery after surgery was often described as a delicate balance between resting and physical activity. The participants who prioritized physical activity and maintained an active lifestyle reported feeling the most recovered. Many emphasized that staying physically and cognitively engaged was crucial to their recovery journey. While some participants reported feeling physically recovered within three months, others continued to struggle with a sense of unsteadiness even a year after surgery, relying on walking aids due to a fear of falling. This insecurity prevented them from resuming more demanding activities, such as skiing or running, and limited their sense of full recovery.

” I tried to ski the other day or maybe a month ago, but I’m terrified of falling and dislocating the joint again, so I don’t dare to. So, there are some limitations.” (Participant 35, 1-year post-surgery).

While many participants felt largely recovered after a year, some faced setbacks from new illnesses or were less active. Lingering physical issues made recovery more complicated for a few. Common problems included muscle pain, vertigo, and worsening osteoarthritis in other joints. Those who stayed consistently active during recovery usually felt better and made more progress.

## Discussion

This study describes the recovery process as a journey along a bumpy road requiring strength, rest and patience to navigate physical, cognitive, emotional and social challenges before reaching a new stable state. It contributes by providing a long-term account of recovery as perceived by older adults one year after hip replacement surgery, an area less commonly explored in previous research.

While earlier studies often focus on short-term outcomes, our findings highlight that recovery continues well beyond the immediate postoperative period, encompassing fluctuating progress and complex personal experiences. A particularly novel finding is the difficulty participants had in distinguishing between postoperative symptoms and those associated with aging, which influenced how they interpreted their recovery and shaped their behaviors. The participants often attributed lingering symptoms to either surgery or aging, reflecting a common belief that illness is a natural part of growing older. Our findings align with prior research showing that those who internalize this stereotype are less likely to engage in health-promoting behaviors such as exercising, maintaining a healthy diet, and seeking medical care [31]. These findings call for age-sensitive support strategies that address both physical and psychosocial aspects and extend into the long-term recovery trajectory.

The participants’ preoperative experiences and expectations influenced how they navigated challenges during the late and long-term stages of recovery. Some participants reported new or worsening symptoms, whereas most experienced a gradual reduction in physical symptoms and cognitive and emotional impacts on daily life. Postoperative recovery in this study is characterized by an initial phase of loss and suffering, followed by a continuous restoration of physical and social functions, which aligns with findings from several other studies [4, 6, 10, 11]. The presence of symptoms has been reported to impact the quality of the recovery transition [32]. Both physical and emotional limitations due to surgery or preexisting conditions play a role in an individual’s ability to manage their recovery, especially their daily life activities [32].

In the late recovery phase, participants reported a wide range of symptoms, such as pain, fatigue, disturbed sleep, and appetite loss. To manage these problems, they adopted coping strategies such as rest, daytime naps, reading, or limiting social interaction. Finding a balance between activity and rest can be challenging, and individuals often seek reassurance that their recovery process follows a typical trajectory [33]. Cognitive difficulties, such as memory decline, added to these challenges, making it difficult for individuals to remember whether they had taken their medication. Even one year after surgery, some participants reported memory loss and ongoing fatigue, although most had comorbidities or other issues affecting their energy levels. Despite these challenges, recovery involved a gradual transition toward improved well-being.

This study suggests that regaining function and independence are crucial parts of the recovery process, which has been explored and clarified by other studies, as participants regain control over physical, social, and habitual factors [6, 10, 12, 34]. Another study indicated that regaining function and strength is essential, and understanding the recovery process is necessary [9]. The participants’ descriptions suggested that their preoperative physical activity levels and degree of social engagement influenced how they experienced and managed the recovery process. These personal resources appeared to facilitate a more proactive and resilient approach to recovery. Previous research has similarly emphasized the importance of reclaiming independence and resuming meaningful personal life activities as central components of the recovery experience [34]. Both physical activity and cognitive engagement emerged as significant themes in participants’ narratives about regaining function and adapting to life after surgery. These findings are supported by previous research showing that physical rehabilitation, along with support from occupational therapists and physiotherapists, is valued by patients and contributes positively to the recovery process [34, 35]. A person’s inability to perform self-care can lead to mixed emotions: they might feel a sense of relief when they successfully address an issue but also experience insecurity when they struggle to do so [32]. These findings highlight the potential value of assessing patients’ preoperative status and offering anticipatory support to facilitate recovery. Informing patients and families that symptoms such as fatigue, mood changes, and memory loss can be part of a normal recovery process may help set realistic expectations and reduce distress [7, 11, 14, 19, 34].

### Strengths and limitations

To the best of our knowledge, this is the first study that provides data capturing the experience and changes in recovery over time among older adults during the first year after hip replacement surgery. The longitudinal qualitative study design enabled a deeper understanding and identification of changes and processes that might have been overlooked in a single-point-in-time study. By conducting individual interviews with participants on two different occasions, a more comprehensive understanding of the recovery process was achieved. The substantial number of participants enabled a rich and varied description of the participants’ journey toward recovery.

This study has several limitations. Some of the interviews were conducted during the COVID-19 pandemic, which may have influenced the results, particularly with respect to social isolation and opportunities for social interaction. These aspects were more prominent in the interviews conducted during the pandemic. Despite the differences in experiences due to the pandemic, the participants were consistent in their statements, and data saturation was achieved. Additionally, the pandemic prolonged the data collection period. Nevertheless, the consistency of having the same interviewer, who followed a structured interview guide, enhanced the study’s dependability. The research team, composed of registered nurses with extensive postoperative care experience, brought expertise to the study. However, this could have led to bias during the analysis and interpretation of the findings. To address this, we used investigator triangulation and held regular meetings and discussions to maintain active reflexivity.

All 60 interviews contributed to the results. Although some were brief, they still contained interesting and valuable insights. While the brevity of certain interviews could be seen as a limitation, the high number of interviews overall strengthens the study. Moreover, the researchers concluded that data saturation had been reached, as no additional aspects of the phenomenon emerged during the analysis of the final interviews.

Another limitation is the lack of patient and public involvement in the study design.

### Recommendations for further research

The findings of this study underscore the considerable variation in recovery experiences among older adults, with some patients recovering more rapidly and fully than others did. A key observation was the role of physical activity and social engagement in enhancing recovery, suggesting that these factors may play a significant role in improving outcomes, but further research is needed to explore these associations in greater depth. Understanding self-care strategies and their impact on recovery is also crucial.

Furthermore, our study highlighted the challenges faced by older adults with extended recovery periods, particularly those with cognitive decline, fatigue, and emotional distress. Future studies should focus on identifying patients who are at greater risk of prolonged recovery and developing targeted interventions to address their unique needs. Specifically, the impact of cognition on recovery warrants further investigation, especially regarding how cognitive decline (e.g., memory loss and concentration issues) affects patients’ ability to engage with their rehabilitation. Additionally, exploring interventions that provide more comprehensive and individualized support for patients, particularly in the early stages post-surgery, could be pivotal in improving recovery outcomes.

Finally, considering the importance of physical and cognitive engagement during recovery, research should also explore how rehabilitation programs can integrate tailored physical activity and cognitive exercises to support a more holistic recovery process.

## Conclusions

This study highlights the complex and individualized nature of the recovery process following hip replacement surgery. The participants navigated a range of physical, cognitive, emotional, and social challenges, ultimately reaching a new stable state one year after surgery. These findings emphasize the importance of understanding the dynamic and multifaceted aspects of recovery, including the influence of preoperative experiences and expectations. The importance of balancing rest and activity, managing symptoms, and maintaining social connections emerged as key factors in the recovery journey. Preoperative education and support from healthcare providers and family members play a vital role in facilitating a smoother transition through recovery phases. Overall, this study contributes to a deeper understanding of older adults’ recovery process, emphasizing the need for personalized care and support to help individuals regain their independence and quality of life after hip replacement surgery. Future research should continue to explore the long-term recovery experiences of older adults to further support and improve postoperative care strategies.

## Supplementary Information

Supplementary Material 1

Supplementary Material 2

Supplementary Material 3

## Data Availability

The datasets used and/or analyzed during the current study are available from the corresponding author on reasonable request.
