# Supplementary material for: The bumpy road to recovery: older adults’ experiences during the first year after hip replacement surgery - a longitudinal qualitative study
Source: BMC Geriatr. 2025 Jul 2;25:483. doi: 10.1186/s12877-025-06155-6 (PMC12220040; doi:10.1186/s12877-025-06155-6)
Supplement: Supplementary file 1 — Supplementary Material 1 [file 12877_2025_6155_MOESM1_ESM.docx]

**Supplementary material**

**Interview guide 14 Days**

Open questions to patients, encouraging them to speak freely about their experiences of recovery, memory and concentration after hip replacement surgery.

- How do you perceive yourself from the time you were cared for in the recovery ward

until now?

- How do you feel since you underwent your hip replacement surgery?
- Do you feel like yourself again after your hip replacement surgery?
- How is your memory after the surgery compared to before?
  - If yes, how and when did you notice this?
  - Can you provide an example?
- Do you experience any changes in your concentration and attention?
  - If Yes could you elaborate?
  - Could you give an example or describe a situation?
- Have you, or do you experience any changes in mood after the surgery?
  - Feel free to elaborate on your answer.
- Can you describe your sleep before and after the surgery?
  - If it has changed, what do you think it might be due to?
  - In what way is your sleep changed?
- Do you feel completely restored to your usual self, regarding your cognitive abilities

after your surgery?

- If not, describe freely in what way and how. Can you describe the process?
- How do you perceive your ability to initiate your daily activities?
  - Can you provide examples and describe it?
- How are your energy levels?

**Probing questions**

- Would you like to elaborate?
- Can you tell me more?
- Can you describe a specific situation?
- Has this changed over time?
- How would you describe the similarities or differences compared to the time before the surgery?
- What has been positive?
- What challenges have you experienced?
- What has helped you?

**Interview guide: 12 months after surgery**

Open questions to patients, encouraging them to speak freely about their recovery, memory, and concentration 12 months after hip replacement surgery.

surgery.

**Main question:**

- How would you describe your recovery period after the surgery?

**Follow-up question:**

- Since our last conversation, have you noticed any changes?
  - If yes, in what way?
  - If no, can you elaborate?

If the patient does not remember what was discussed in the previous interview, the interviewer summarizes the key points and then asks how the patient would describe their current situation in relation to that summary.

**Probing questions**

- Would you like to elaborate?
- Can you tell me more?
- Can you describe a specific situation?
- Has this changed over time?
- How would you describe the similarities or differences compared to the time before the surgery?
- What has been positive?
- What challenges have you experienced?
- What has helped you?
