# Supplementary material for: The bumpy road to recovery: older adults’ experiences during the first year after hip replacement surgery - a longitudinal qualitative study
Source: BMC Geriatr. 2025 Jul 2;25:483. doi: 10.1186/s12877-025-06155-6 (PMC12220040; doi:10.1186/s12877-025-06155-6)
Supplement: Supplementary file 2 — Supplementary Material 2 [file 12877_2025_6155_MOESM2_ESM.pdf]

Creating Connections.
Changing Lives.

April 1, 2025

Ulrica Nilsson | Professor, PhD, RNA, FAAN
Karolinska Insitutet
Alfred Nobels Allé 23
141 52 Huddinge
Sweden

RE: Research studies titled, *Patients Experiences of Early Postoperative Cognition (COME)* **and** *Evaluation of a digital, self-administered, neurocognitive test battery in older adult patients undergoing abdominal surgery – a non-randomized feasibility trial*

Dear Dr. Nilsson:

This letter is to confirm receipt of payment for your unauthorized use of an unauthorized version of the Swedish MMSE in the research studies referenced above (Invoice IN-00445196).

Also by way of this letter, you may use the data obtained from said research to publish the results of these studies, subject to the following conditions:

1) The publication(s) **must not include** any test items from the unauthorized version of the Swedish MMSE that was used without permission, or any other version of the MMSE in any language.

2) The publication(s) **must include** this statement:

   An unauthorized version of the Swedish MMSE was used by the study team without permission, however this has now been rectified with PAR. The MMSE is a copyrighted instrument and may not be used or reproduced in whole or in part, in any form or language, or by any means without written permission of PAR (www.parinc.com).

3) One (1) copy of each article must be sent via email to PAR to confirm that the above conditions have been met.
   Email: vmark@parinc.com

If you have any questions or concerns, please do not hesitate to contact me directly.

Sincerely,

Vicki M. McFadden
Vicki M. McFadden
Sr. Permissions Specialist
vmark@parinc.com
